# Clinical and pathological correlates of severity classifications in trigger fingers based on computer-aided image analysis

**DOI:** 10.1186/1475-925X-13-100

**Published:** 2014-07-23

**Authors:** Tai-Hua Yang, Hsin-Chen Chen, Yung-Chun Liu, Hui-Hsuan Shih, Li-Chieh Kuo, Stephen Cha, Hsiao-Bai Yang, Dee-Shan Yang, I-Ming Jou, Yung-Nien Sun, Fong-Chin Su

**Affiliations:** 1 Department of Biomedical Engineering, National Cheng Kung University, 1 University Road, Tainan 701, Taiwan; 2 Department of Computer Science & Information Engineering, National Cheng Kung University, 1 University Road, Tainan 701, Taiwan; 3 Department of Pathology, National Cheng Kung University, Tainan 701, Taiwan; 4 Department of Occupational Therapy, National Cheng Kung University, Tainan 701, Taiwan; 5 Medical Device Innovation Center, National Cheng Kung University, Tainan 704, Taiwan; 6 Department of Orthopedics, National Cheng Kung University, Tainan 701, Taiwan; 7 Department of Radiation Oncology, Washington University in Saint Louis, Saint Louis, MO 63110, USA; 8 Department of Health Science Research, Division of Biomedical Statistics and Informatics, Mayo Clinic, Rochester, MN 55902, USA; 9 Orthopedic Biomechanics Laboratory, Division of Orthopedic Research, Mayo Clinic, Rochester, MN 55905, USA; 10 Department of Pathology, Ton-Yen General Hospital, Hsinchu 302, Taiwan; 11 Department of Orthopedic Surgery, Ton-Yen General Hospital, Hsinchu 302, Taiwan; 12 Department of Orthopedics, China Medical University Hospital, Taichung 404, Taiwan

**Keywords:** Severity correlation, Computer-aided, Image analysis, Pulley pathology, Trigger finger

## Abstract

**Background:**

The treatment of trigger finger so far has heavily relied on clinicians’ evaluations for the severity of patients’ symptoms and the functionality of affected fingers. However, there is still a lack of pathological evidence supporting the criteria of clinical evaluations. This study’s aim was to correlate clinical classification and pathological changes for trigger finger based on the tissue abnormality observed from microscopic images.

**Methods:**

Tissue samples were acquired, and microscopic images were randomly selected and then graded by three pathologists and two physicians, respectively. Moreover, the acquired images were automatically analyzed to derive two quantitative parameters, the size ratio of the abnormal tissue region and the number ratio of the abnormal nuclei, which can reflect tissue abnormality caused by trigger finger. A self-developed image analysis system was used to avoid human subjectivity during the quantification process. Finally, correlations between the quantitative image parameters, pathological grading, and clinical severity classification were assessed.

**Results:**

One-way ANOVA tests revealed significant correlations between the image quantification and pathological grading as well as between the image quantification and clinical severity classification. The Cohen’s kappa coefficient test also depicted good consistency between pathological grading and clinical severity classification.

**Conclusions:**

The criteria of clinical classification were found to be highly associated with the pathological changes of affected tissues. The correlations serve as explicit evidence supporting clinicians in making a treatment strategy of trigger finger. In addition, our proposed computer-aided image analysis system was considered to be a promising and objective approach to determining trigger finger severity at the microscopic level.

## Introduction

Trigger finger (Stenosing Tenovaginitis) is one of the most common hand diseases. The name for this condition was first proposed by Notta in 1850 [1,2]. In the general prevalence of trigger finger, the age distribution showed a bimodal shape with one group below 6 years of age and the other above 40 years of age. Especially among adults, women between 50 and 60 years old were the most common population to experience this disease [3]. Most cases involved a single finger, but multiple fingers were affected in some patients. The thumb or ring fingers were the most commonly affected digits. Furthermore, the right side was more frequently affected than the left [3,4]. There were no dependable symptomatic patterns of the trigger finger. The symptoms developed gradually. There were many causative factors, such as activities consisting of numerous power grip or repetitive performances, trauma, congenital anatomic abnormality, etc., but more were idiopathic. Trigger finger was generally diagnosed at the location of the first annular (A1) pulley due to the narrowing of the canal or bulging of the tendon, conditions which restricted the normal gliding of the tendon. Symptoms included pain, disability, snapping, or even locking of the fingers in a flexed position. This locking occasionally required passive manipulation of the finger into a fully extended position. Sometimes, a secondary joint contracture in a flexed position eventually developed [3,4]. Many taxonomic systems were reported in order to diagnose the condition and distinguish the severity of the symptoms.

According to these clinical classifications, physicians could make medical decisions easily. Those bases of classifications were graded by symptoms (e.g., pain and triggering) or/and mechanical problems (e.g., locking and joint contracture). Newport et al. in 1990 [5] and Froimson in 1999 [6] proposed different classification systems according to mechanical disorders and symptoms. Quinnell and Eastwood et al. sorted them based on severity of symptoms [7,8], and Patel and Bassini graded them with mechanical disorders only [9]. To date, no effective and uniform clinical classification exists. Even less work has been done in attempting to correlate clinical severity classifications and pathological grading. Drossos et al. described and demonstrated correlations between the histological abnormalities in pathological A1 pulleys and the clinical severity classification system developed by Newport et al. [10]. However, Drossos et al. staged the pathological severity into 3 grades according to histopathological findings by expert pathologists.

So far, the main approach in interpreting abnormalities in pulley microscopic images and grading the stages of trigger finger remains the visual evaluation by pathologists. Such a method, however, is highly dependent of subjective judgments, theoretical knowledge and interpreting experience [11], and thus produces inaccurate results and irreproducible evaluation results [12]. These limitations might decrease the accuracy of evaluating the stage for the final diagnosis.

With the rapid development of image processing techniques, digital image analysis has been used widely for systemic survey and investigation in many disciplines. Digital analysis provides the most direct information, not only from gross texture, but also from tissue or cells at the microscopic or biomolecular level, which may have morphological or feature changes [12-21]. In this study, we employed an automatic quantitative image analysis system developed by Liu et al. in 2013 [16] in order to create a quantitative, accurate and reproducible assessment method. With this system, we attempted to address three correlations through a quantitative experimental study. First, we investigated the correlation between (1) the automatic quantitative microscopic digital image evaluation and (2) pathological grading to investigate quantitatively accuracy of pathological characteristic analysis by digital image analysis system. Then, we investigated the correlation between the automatic quantitative digital image evaluation and (3) clinical severity classification which applied Froimson’s classification grade II to IV. With these correlations analyzed, we were able to derive the correlation between the quantitative pathological grading and the quantitative clinical severity classification.

## Materials and methods

### Materials

All microscopic images of acquired trigger finger with written informed consents from patients were provided by National Cheng Kung University Hospital and Ton-Yen General Hospital. All patients were informed about the aims of the study and signed consent forms with detailed descriptions regarding the acquirement of their microscopic images. The study was approved by the Institutional Review Board of National Cheng Kung University Hospital and Ton-Yen General Hospital. A total of 21 patients who had been diagnosed with acquired trigger finger by two experienced orthopedists and had received an open release operation were included in this study. Patients with congenital trigger finger, corticosteroid injection, known hand tumor or deformity, rheumatoid arthritis, inflammatory arthritis, flexor tendinitis, hemodialysis, sarcoidosis, diabetes mellitus, metabolic disorder, amyloidosis, or major trauma (fracture or ligament tear) of the ipsilateral arm were excluded. The severity of trigger fingers were graded by Froimson in 1999 [6] in clinical assessment as followed:

•*Grade I* “Pre-triggering-pain; tenderness over the A1 pulley; history of catching but not demonstrable on physical examination”.

•*Grade II* “Triggering, active; demonstrable catching, patient can actively extend”.

•*Grade III* “Triggering, passive; demonstrable catching requiring passive extension or inability to actively flex”.

•*Grade IV* “Contracture; demonstrable catching, with a fixed flexion PIP joint contracture”.

The surgical indication included clinical severities of grades III and IV and grade II patients whose symptoms persisted after 3 months of conservative treatment.

Briefly, all surgical procedures were performed under local anesthesia in this study. Once the anesthetic took effect, the skin of the operative area was sterilized with antiseptic fluid and draped with surgical towels. A 1.5-cm longitudinal incision was made in the skin over the affected A1 pulley. To protect the digital nerves and vessels, the subcutaneous tissue and palmar fascia were dissected bluntly, and the thickened A1 pulley was carefully identified. A 0.5-cm-wide full-thickness section of the A1 pulley from the proximal to distal edge was excised longitudinally. The wound was closed with absorbable sutures and the specimens were then sent for pathological analysis. For pathological examination, all of the specimens were processed according to standard procedures: fixation in 10% formalin, procession in graded alcohols and xylene for dehydration, spreading of tissue flat and embedding it in paraffin, cutting of 5-μm semi-thin sections transversely and longitudinally with a microtome, and staining for light microscopy with hematoxyline-eosin (H&E) for future analysis.

**Figure 1:**
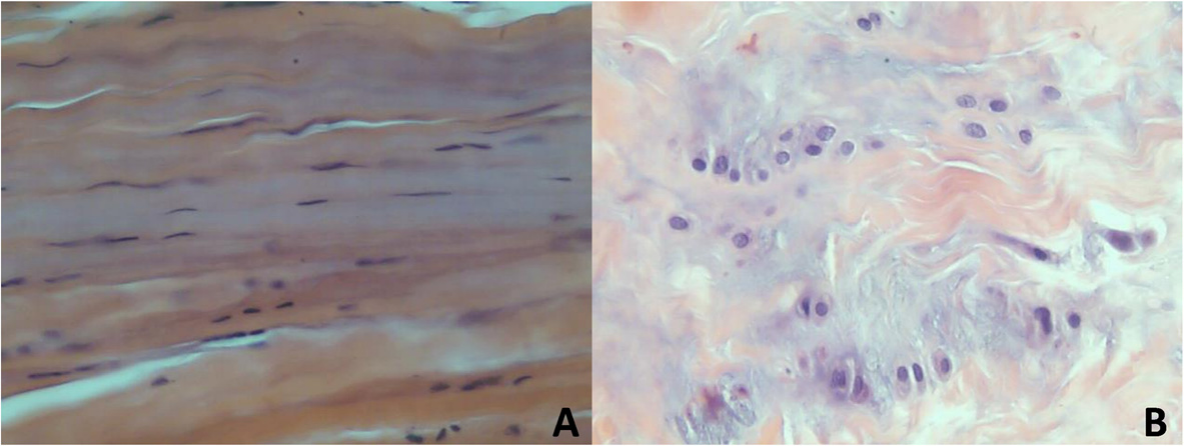
**Features of A1 pulley in normal and trigger finger. (A)** Normal A1 pulley tissue with pink collagen deposits and fibrocytes with elongated nuclei; **(B)** Trigger finger A1 pulley tissue, presence of chondroid metaplasia with a blue-color chondromyxoid matrix and rounded nuclei of chondrocytes.

Generally, the normal pulley (Figure 1A) was composed of dense compact and parallel regular collagenous fibrotic bundles that appeared eosinophilic pink and rows of modified fibroblasts with elongated spindle-shaped nuclei between the bundles. In contrast, the pathologic pulley (Figure 1B) was composed of irregular connective tissue with chondroid metaplasia (or fibrocartilaginous metaplasia), which exhibited more chondromyxoid materials and showed basophilic blue or purple colors because large amounts of hyaluronic acid, chondroitin sulfate and proteoglycan accumulated [10,22-24]. Furthermore, nuclei of cartilage-like cells had become rounded in shape [23]. All the specimens used in the study were examined by three pathologists and sorted into 3 grades [16] according to a modified version of the description proposed by Sbernardori and Bandiera in 2007 [23] and classification proposed by Drossos et al. in 2009 [10]. The low grade (L) included those with a slightly basophilic myxoid matrix between collagen bundles. The middle grade (M) showed irregular distribution of a chondromyxoid matrix and some sporadic round cartilaginous cells. The high grade (H) revealed patches of a chondromyxoid matrix and an increase of clustered round cartilaginous cells called chondroid metaplasia [16].

### Methods

Seven male (33%) and fourteen female (67%) patients with an average age of 55.7 ± 13.3 years (a range of 25-75 years) were included in this study. Pathological pulley specimens came from 19 right hands (90%) and 2 left hands (10%) with 12 long fingers (57%), 7 thumbs (33%) and 2 ring fingers (10%). In clinical severity classification, there were 8 in grade II, 6 in grade III and 7 in grade IV according to Froimson’s classification system [6]. In pathological grading, there were 5 in grade L, 8 in grade M, and 8 in grade H [16].

For each specimen, 49 images with a size of 2560 × 1920 were acquired through the use of a developed auto-focusing whole slide images system [16,25] (Nikon Eclipse 50i microscope with Nikon Ds-Fi1 5-megapixel CCD camera and Prior OptiScan-II scanning platform). Then the same pathologists involved in this study were in charge of finding and discarding unsuitable images based on their knowledge of tissue pathology. These images contained only a small number of nuclei and large areas of background and irrelevant tissues (e.g., microvasculature), therefore providing rare evidence of pulley tissues. Thus, they were unsuitable for validating the relationship between the proposed image parameters and trigger finger disease. Most of the suitable images came from the thicken part in the middle section of the specimens. Afterward, a random selection process was performed to acquire 10 images from the remaining image samples of each specimen. These images presented sufficient contents of pulley tissues for the quantitative parameter analysis.

**Figure 2:**
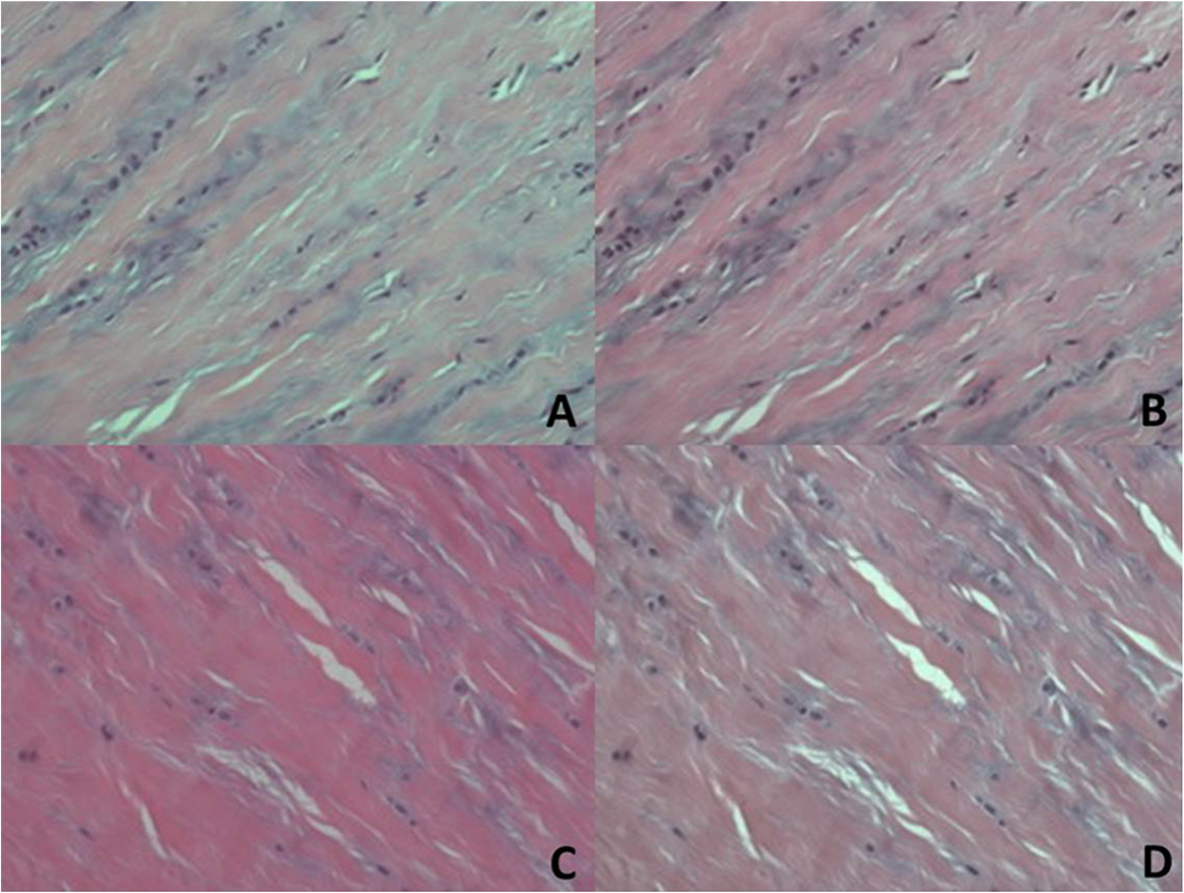
**Color normalization.** Original image **(A)** and **(C)**; Normalized results **(B)** and **(D)**, respectively.

**Figure 3:**
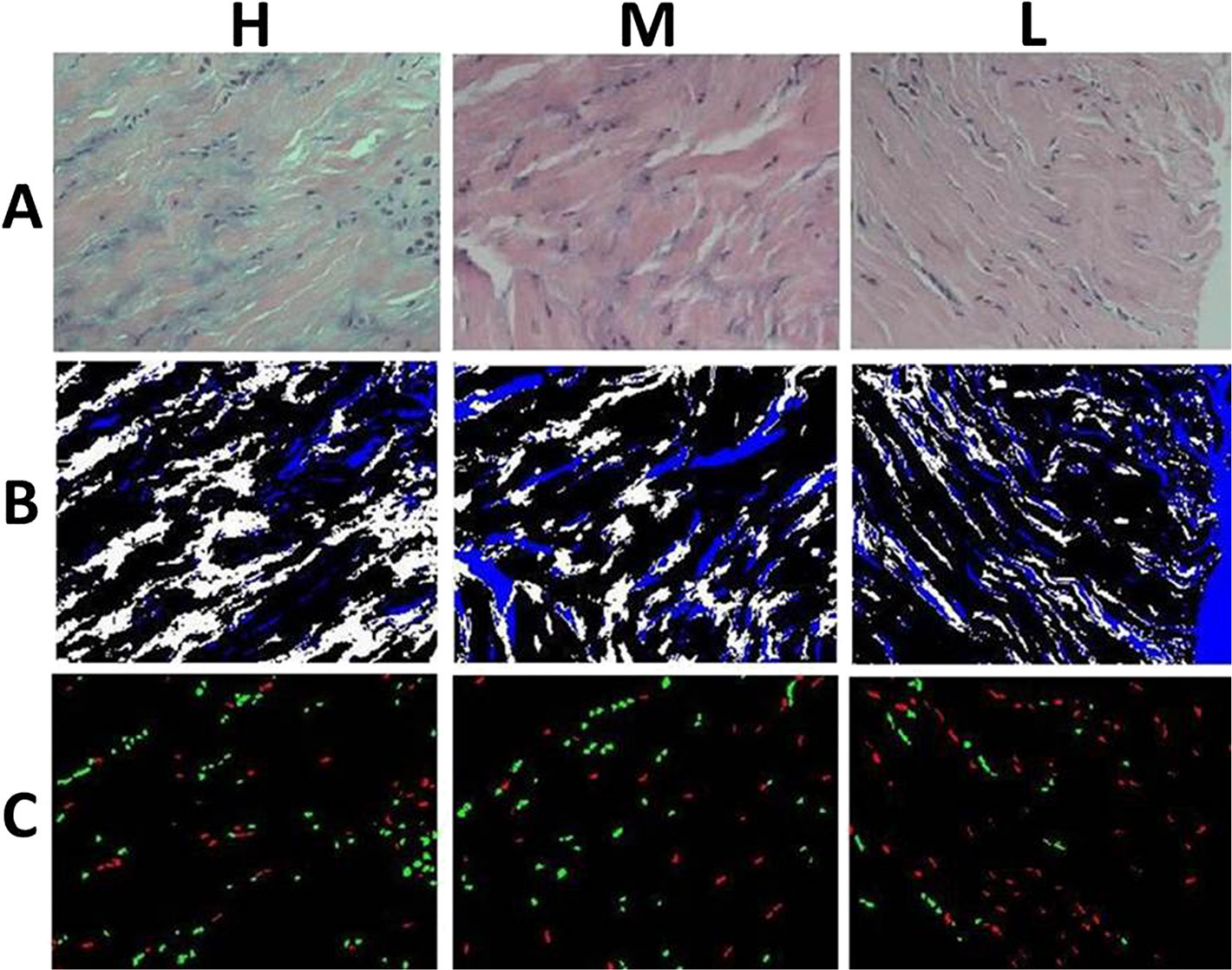
**Results in different steps of processing. (A)** The normalized images of the three pathological grades. **(B)** The color segmentation (white: abnormal tissue region, black: normal tissue region, blue: empty background). **(C)** The nuclei classification (red: normal, green: abnormal).

To quantify the image parameters objectively, we employed our previously developed image analysis system [16] which contained four automatic processing steps as follows: The first step was color normalization (Figures 2 and 3A). This method was used to resolve the problem of non-uniform distribution of color and illumination in the acquired images. Such inconsistencies were caused by the different staining and imaging conditions of the microscopic slices. The second step was a three-stepped color segmentation process. This step separated and labeled the background (blue), normal tissue regions (black) and abnormal tissue regions (white) (Figure 3B). The third step was nuclei classification. This step used an active double-thresholding scheme to distinguish normal (red) and abnormal (green) nuclei (Figure 3C). The fourth step was the computation of the parameters of the ratios of abnormal regions and nuclei for severity evaluation. Two parameters can be automatically calculated based on the color appearance of the matrix and the shape characteristics of the nuclei on the selected slides. Parameter 1 (Abn-SR) indicated the size ratio of the abnormal tissue region, which was calculated by dividing the area of the abnormal tissue region by the total area of the tissue region (normal and abnormal). Parameter 2 (Abn-NR) indicated the number ratio of the abnormal nuclei, which was calculated by dividing the number of abnormal nuclei by the total number of nuclei (normal and abnormal). According to our previous research [16] and the analysis which used bivariate analysis to illustrate the relationship between the disease stages, the resulting correlation coefficient of the two parameters was 0.7484. Therefore, this calculation denoted they are positively and highly correlated.

### Statistics

All measurements were expressed as the mean and SD. In this study, box-and-whisker plots [26] were used to describe all the parameters of data distribution from these severity groups of pathological grading and clinical severity classification through their quartiles. The lines (whiskers) extending vertically from the boxes indicated variability outside the upper and lower quartiles. Moreover, outliers were plotted as distinct points. The outcome measurements were analyzed using a one-way analysis of variance (ANOVA). Post hoc pairwise comparisons were performed under the least significant difference (LSD) rule if there was a significant difference. Any *p*-value less than 0.05 were considered as statistically significant. Cohen’s kappa coefficient was applied to measure the agreement between these two grading systems. The result was interpreted as follows: (1) <0.40 was rated “poor” (2) 0.40-0.74 was “fair to good” (3) 0.75-1.0 was “excellent”. All statistical analyses were performed by JMP version 9.01 (SAS institute Inc. Cary, NC, USA).

## Results

**Figure 4:**
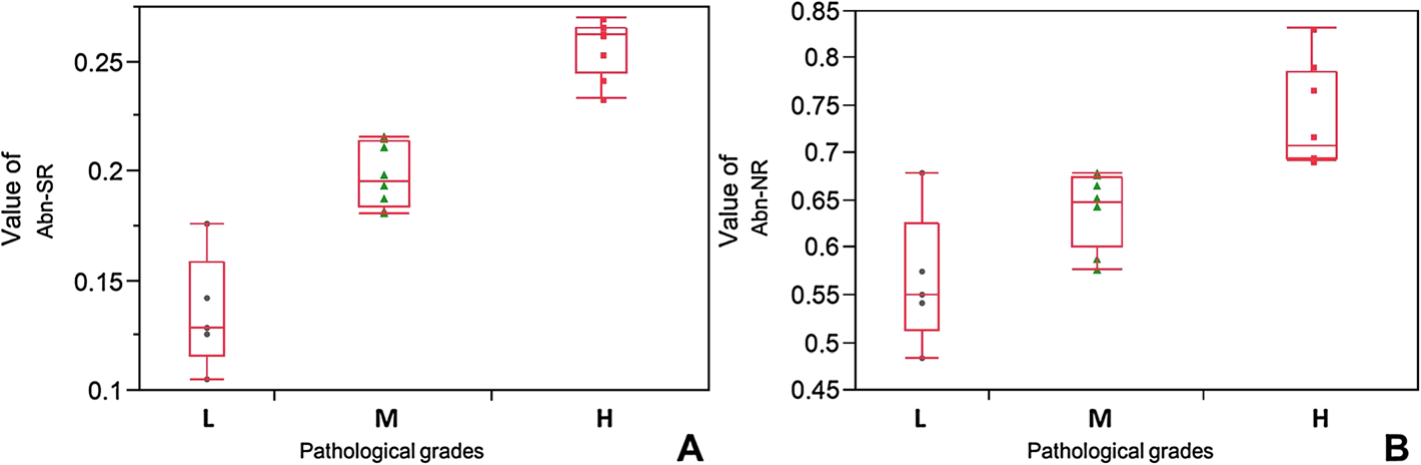
**Analysis of two parameters in pathological grades. (A)** Ratio of abnormal region (Abn-SR, *p* < 0.0001). **(B)** Ratio of abnormal nuclei (Abn-NR, *p* < 0.0001).

**Figure 5:**
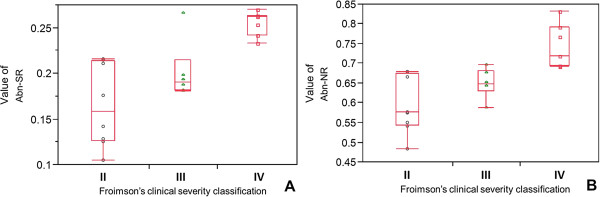
**Analysis of two parameters in Froimson’s clinical severity classification. (A)** Ratio of abnormal region (Abn-SR, *p* = 0.0003). **(B)** Ratio of abnormal nuclei (Abn-NR, *p* = 0.0005).

The mean values of these two parameters were calculated from the images of the subjects, whose pathological grades and clinical severity classifications were specified by the pathologist and orthopedist, respectively, as listed in Table 1. The mean values of the ratio of abnormal region (Abn-SR) relative to pathological grading (L, M and H) were 0.14 ± 0.03, 0.20 ± 0.01 and 0.26 ± 0.01, respectively. The mean values of the ratio of abnormal nuclei (Abn-NR) relative to pathological grading (L, M and H) were 0.57 ± 0.07, 0.64 ± 0.04 and 0.74 ± 0.05, respectively. The mean values of Abn-SR matching Froimson’s clinical severity classifications (II, III and IV) were 0.16 ± 0.05, 0.20 ± 0.03, and 0.26 ± 0.01, respectively, and the mean values of Abn-NR for the three classifications were 0.59 ± 0.07, 0.65 ± 0.04, and 0.74 ± 0.06, respectively. The boxplots of the two parameters according to pathological grading were plotted in Figure 4, and those according to clinical severity classification were plotted in Figure 5. The one-way ANOVA revealed that both Abn-SR and Abn-NR had significant differences among the three pathological grades (Abn-SR: *p* < 0.0001, Abn-NR: *p* < 0.0001). Moreover, the one-way ANOVA between the three clinical severity classifications also revealed significant differences (Abn-SR: *p* = 0.0003 and Abn-NR: *p* = 0.0005). Comparisons for each pair between different pathological grades and clinical severity classifications calculated with Post hoc pairwise comparisons under LSD rule are shown in Table 2, respectively. All pairwise *p*-values showed a significant difference except the pairs of Froimson’s classification grade II to III in both parameters. Furthermore, percentage distribution and contingency analysis of Froimson’s clinical severity classifications by pathological grading are shown in Table 3. These data were used to calculate the Cohen’s kappa coefficient. The Cohen’s kappa coefficient 0.717 represented good substantial agreement between pathological grades and clinical severity classifications.

**Table 1:** Mean Abn-SR and Abn-NR in pathological grades and in Froimson’s clinical severity classification respectively

	**Pathological grade**			**Froimson’s clinical severity classification**		
	**L grade**	**M grade**	**H grade**	**Grade II**	**Grade III**	**Grade IV**
**Abn-SR**	0.14 ± 0.03	0.20 ± 0.01	0.26 ± 0.01	0.16 ± 0.05	0.20 ± 0.03	0.26 ± 0.01
**Abn-NR**	0.57 ± 0.07	0.64 ± 0.04	0.74 ± 0.05	0.59 ± 0.07	0.65 ± 0.04	0.74 ± 0.06

*Abbreviations*: *Abn-SR* ratio of abnormal region, *Abn-NR* ratio of abnormal nuclei.

**Table 2:** ***p*-value of Post hoc pairwise comparisons under LSD rule between pathological grades and between clinical severity classifications respectively**

	**Comparison of pathological grades**			**Comparison of Froimson’s clinical severity classification**		
	**H - L**	**M - L**	**H - M**	**IV - II**	**IV - III**	**II - III**
**Abn-SR**	<0.0001*	<0.0001*	<0.0001*	<0.0001*	0.0104*	0.0602
**Abn-NR**	<0.0001*	0.0242*	0.0022*	<0.0001*	0.0123*	0.0931

*Abbreviations*: *Abn-SR* ratio of abnormal region, *Abn-NR* ratio of abnormal nuclei; *, significant difference.

**Table 3:** Distribution and contingency analysis of clinical severity classification according to pathological grades (Cohen’s kappa coefficient = 0.717)

		**Froimson’s clinical severity classification**			
		**Grade II**	**Grade III**	**Grade IV**	**Total**
**Pathological grade**	L grade	5 (100%)	0	0	5
	M grade	3 (37.5%)	5 (62.5%)	0	8
	H grade	0	1 (12.5%)	7 (87.5%)	8
Total		8 (38.1%)	6 (28.6%)	7 (33.3%)	21

In addition, the inter-observer variability for clinical severity classification and pathological grading of trigger fingers was evaluated using an interclass correlation coefficient (ICC) [27]. The ICC for the clinical classification results of the two orthopedists was 0.983, and the ICC for the pathological grading of the three pathologists was 0.979.

## Discussion

From the clinician’s perspective, the strategies and plans of treatment are decided based on the severities of trigger finger disease determined by the patient’s clinical symptoms and physical examination performance. There was, however, a lack of an objective, quantitative, reliable and consistent grading system till now. In addition, the conformity between pathological and clinical severity has rarely been explored and discussed. Drossos et al. first proposed three pathological severity grades of trigger finger based on the analysis of microscopic images. They also compared the pathological severity grades with the clinical severity classification [10]. But those diagnoses were all achieved through the human interpretation of some episodes in the microscopic images instead of comprehensive surveys and contained much human bias. Furthermore, Tung et al. proposed a series of quantitative evidence between impaired trigger finger severities and quantitative kinematic characteristics [28]. This study demonstrated the workspace of the hand, the range of motion and finger joints and the ratio of angular acceleration range between the extension and flexion of finger joints. However, their study addressed clinical grading by the use of kinematic evidence rather than the pathological changes associated with different clinical severities.

Diagnostic pathology is an important and integral discipline. It provides, not only the final diagnosis of a disease, but also the differences between the levels of severity of a disease through the identification of tissue features from microscopic images. The results of diagnosis are usually affected by the vision, knowledge, and experience of the pathologist and the quality of the slices and images. In this study, we utilized our developed automatic image analysis system [16] to measure the two major microscopic imaging parameters (Abn-SR and Abn-NR) as mention above from the A1 pulley trigger finger specimen. The pathological findings of trigger finger were explored and described with respect to the obvious changes in the inner layer (gliding surface) and the obvious increase of chondroid metaplasia with chondromyxoid degeneration, rounded nuclei of chondrocytes and hypervasculization [10,22,23]. But in the automatic image analysis system used in this study [16], the computerized grading system was focused on assessing the changes in the first two pathological categories (without hypervasculization). This grading system differed from the system of Drossos et al. [10]. In this study, the results showed that changes in color and shape can be automatically and efficiently identified from the image features. With the use of the automatic image analysis system, human errors caused by physical factors and subjective experience can be avoided. This study demonstrated a high positive correlation between increased chondroid metaplasia and cell density on the one hand and increased severity of trigger finger as defined by pathological grading on the other. The results corresponded with those of previous studies [10,22,23]. Besides, this system also provided accurate discrimination of different clinical severities of Froimson’s grades II, III and IV. Although Post hoc pairwise comparisons under LSD rule revealed no significant difference of parameters between grades II and III (p = 0.0602 for Abn-SR and *p* = 0.0931 for Abn-NR), nevertheless, a trend to distinguish them was evident. The lack of significant difference between grades II and III may be related to the individual responses of patients in examination. While fingers in both classifications were mechanically entrapped due to a mismatch between the pulley canal and bulged tendon in pathophysiology [3], grade II was assigned when the patient could actively extend the finger, and grade III was assigned when the patient had to use the other hand to move the finger passively [6]. However, a number of cases were difficult to classify because of patient performance in examination. Different patients with the same severity of trigger finger might have chosen to move the finger actively or passively depending on their pain tolerance. Moreover, box-and-whisker plots allowed the easy separation of results from the calculation of Abn-SR and Abn-NR into clusters that could be correlated with different severities of pathological and clinical classifications. For the pathological grading system, this automatic image analysis system was able to define effectively these three grades in accordance with the results of Liu et al. [16]. In this study, the strength of agreement with the Cohen’s kappa coefficient revealed good consistency (i.e., correlation) between pathological grades and clinical severity classifications. Therefore, the use of automatic digital image analysis to quantify these two parameters helps to distinguish the severity grades in pathological and clinical classifications. From these results it can be inferred that these two parameters accurately reflected the severity of acquired trigger finger and may have been affected by the change of the material properties of the A1 pulley. As to the inter-observer variability, the ICC values for both the orthopedists and pathologists were higher than 0.95, which is much greater than the 0.75 threshold indicating an excellent consistency of classification [27].

Small sample size was the one of the limitations. In the future, we plan to increase the number of cases and include more clinicians and pathologists to account for differences in individual clinicians’ and pathologists’ judgment and procedures. In addition, the pathological grades were compared only with the clinical classifications that were surgical indications. This included grades III and IV and the grade II patients who had responded poorly to conservative treatment for 3 months. Grade I and some grade II patients can be generally treated conservatively (e.g., drugs, rehabilitation, immobilization and steroid injection) in the clinic. Therefore, we could not easily obtain tissue samples from mild grade trigger fingers.

## Conclusions

The histopathological characteristics of trigger finger are distinctive and form the basis of diagnosis by pathologists. In this study, we first assessed the pathological changes of color in the matrix and shape of the nuclei in images of trigger fingers by applying our customized automatic image analysis system. This investigation revealed that chondroid metaplasia (or fibrocartilaginous metaplasia) varied with the severity of trigger finger, showing an increase in blue-colored chondromyxoid matrix and round-shaped chondroid nuclei. Since these two pathological features were useful for defining the severity of trigger finger, the ratio of abnormal area (Abn-SR) and the ratio of abnormal nuclei (Abn-NR) were then calculated and tested as indices for evaluation of the severity of trigger finger through the image analysis system. The results confirmed the reliability of these indices. Furthermore, this study showed high correlations between the image parameters and pathological grading as well as between the image parameters and clinical classification. More importantly, these quantitative indices have enabled correlations to be made between pathological grading and clinical severity classification. This study not only proves that digital image analysis is useful in the medical field for systemic survey and efficient investigation of larger image samples. It also shows that image analysis system is reliable, helpful and efficient in providing the most direct information and quantitative analysis (compared to human observation) for evaluating morphological or feature changes of pulley tissues at the microscopic level of trigger fingers. With this objective information, the clinician can make a more evidential diagnosis of the severity of trigger finger. Based on this diagnosis, the clinician can then use the most appropriate treatment strategy for the patient.

## Competing interest

The authors declare that they have no competing interest.

## Authors’ contributions

Conceived and designed the experiments: THY, HCC, YCL, HHS, LCK, HBY, IMJ, YNS, FCS. Performed the experiments: THY, YCL, HHS, HBY, DSY. Analyzed the data: THY, YNS, HCC, LCK, SC. Contributed reagents/materials/analysis tools: SC, IMJ, YNS, FCS. Wrote the paper: THY, HCC, YCL, YNS, SC. All authors have read and approved this manuscript.
